# Ergot Alkaloids in Fattening Chickens (Broilers): Toxic Effects and Carry over Depending on Dietary Fat Proportion and Supplementation with Non-Starch-Polysaccharide (NSP) Hydrolyzing Enzymes

**DOI:** 10.3390/toxins9040118

**Published:** 2017-03-28

**Authors:** Sven Dänicke

**Affiliations:** Institute of Animal Nutrition, Friedrich-Loeffler-Institute (FLI), Federal Research Institute for Animal Health, Bundesallee 50, 38116 Braunschweig, Germany; sven.daenicke@fli.de; Tel.: +49-531-5804-4101

**Keywords:** fattening chicken, ergot, carry over, non-starch-polysaccharide, feed enzyme, dietary fat

## Abstract

Ergot alkaloids (EA) are mycotoxins produced by *Claviceps purpurea*. EA-toxicity is poorly characterized for fattening chickens. Therefore, a dose–response study was performed to identify the lowest, and no observed adverse effect levels (LOAEL and NOAEL, respectively) based on several endpoints. Non-starch-polysaccharide (NSP) cleaving enzyme addition and dietary fat content were additionally considered as factors potentially influencing EA-toxicity. Feed intake was proven to respond most sensitively to the EA presence in the diets. This sensitivity appeared to be time-dependent. While LOAEL corresponded to a total dietary EA content of 5.7 mg/kg until Day 14 of age, it decreased to 2.03 mg/kg when birds were exposed for a period of 35 days. Consequently, NOAEL corresponded to an EA content of 2.49 mg/kg diet until Day 14 of age, while 1.94 mg/kg diet applied until Day 35 of age. Liver lesions indicating enzyme activities in serum were increased after 14 days of exposure. Dietary fat content and NSP-enzyme supplementation modified EA toxicity in an interactive manner. The EA residues in serum, bile, liver and breast meat were <5 ng/g suggesting a negligible carry over of intact EA.

## 1. Introduction

*Claviceps purpurea* is a phyto-pathogenic fungus which predominantly infects rye, triticale and wheat and forms a hardened sclerotium known as ergot which develops on the ears in place of a grain. Ergot alkaloids (EA) as the mycotoxins of *C. purpurea* are the main toxic constituents in ergot varying tremendously between nearly zero to approximately 10,400 mg/kg ergot (=1.04%) depending on geographic region, harvesting year, cereal species, variety and genotype [1,2,3]. Despite this variation, the current upper limits for feedstuffs still rely on the mass proportion of ergot in unground cereal grains and are set to 1000 mg ergot (*C. purpurea*) per kg unground cereal grains as specified by Directive 2002/32/EC. The European Food Safety Authority (EFSA) addressed these drawbacks and suggested substituting this inappropriate physical method by chemical analysis as a basis for an animal health-based risk management [4]. Therefore, both the monitoring of feedstuffs for EAs and the setting of upper limits on a similar basis are required. However, both lowest observed adverse effect levels (LOAEL) and no observed adverse effect level (NOAEL) based on EAs are poorly defined for poultry species so far. Therefore, dose–response experiments are required to titrate critical dietary EA concentrations in feed and to derive LOAEL and NOAEL. 

Furthermore, toxic effects of mycotoxins and consequently their critical concentrations in feed might be influenced by nutritional factors. Especially those dietary factors are of interest which markedly modify physico-chemical chyme conditions in such a way that nutrient and possibly toxin absorption is modulated. Non-starch-polysaccharides (NSP) are plant cell wall components which cannot be hydrolyzed by endogenous enzymes and which give rise to an increase in the viscosity of small intestinal chyme especially of growing fattening chickens (broilers) (e.g., [5]). The viscosity increasing potential of NSP is closely associated with its water-soluble part, and rye, wheat and barley are cereal grains characterized by specifically high contents of soluble NSP. Based on the knowledge that diets with higher concentrations of soluble NSP potentially increase the viscosity of small intestinal chyme and decrease nutrient digestibility and absorption, commercial broiler diets are usually supplemented with exogenous NSP-hydrolyzing enzymes which prevent the increase in intestinal viscosity and the adverse down-stream effects. Thus, it can be hypothesized that wheat- and rye-based broiler diets which are not supplemented with NSP-hydrolyzing enzymes would partly prevent the toxicity of EA mediated through an increased intestinal viscosity associated with a lower rate of absorption of nutrients and toxins, while enzyme addition would result in opposite effects.

Another nutritional factor potentially modifying EA toxicity is dietary fat. Compared with other farm animals kept for fattening purposes broiler diets require relatively high fat contents to meet the energy requirement of the animals. Such high-fat diets are characterized by a high digestibility of dietary fat. A high fat digestion and absorption of fatty acids might also facilitate the absorption of EA, especially of those with a higher lipophilicity as recently discussed [6]. Therefore, it might be hypothesized that toxicity of EA is more pronounced after feeding high-fat diets compared to low-fat ones. 

Based on the discussed need for dose–response experiments it was the aim of this experiment to test toxic effects in broilers fed increasing concentrations of EA both in low- and high-fat diets, prepared either without or with NSP-hydrolyzing enzymes.

## 2. Results

### 2.1. Chemical Composition of Ergot, Feedstuffs and Diets

Total EA content of ergot amounted to approximately 0.02% whereby ergotamine, ergocristine and ergosine accounted for 25, 22 and 10% of total EA, respectively (Table 1).

Ergot contained approximately 31% fat which was mainly composed of palmitic acid, oleic acid, linoleic acid and most notably of the ergot-specific ricinoleic acid (approximately 17%). 

As the crude fiber content of approximately 26% not only contained cell wall constituents a detailed NSP-analysis of the ergot was also performed and revealed a total NSP content of 9.5% from which approximately 10% were soluble (Table 2). This approximates the total and soluble NSP content of wheat while rye contained both a higher total NSP content and a higher proportion of soluble NSP. With regard to the individual monomer sugars it is worth noting that wheat and rye were mainly composed of arabinose, xylose and glucose while ergot contained only traces of these sugars. In contrast, ergot contained mainly mannose, galactose and glucose (Table 2). 

The analyzed crude protein contents of the different diets varied between approximately 21% and 22% and consequently matched the targeted similarity (Table 3). Moreover, the intended graduation in EA contents was also achieved, which is supported by the strong linear relationship between calculated and analyzed EA-contents of the diets: *y* = 0.93*x* + 0.12 (*r*^2^ = 0.99). 

### 2.2. Clinical Signs and Pathological Findings

**Figure 1:**
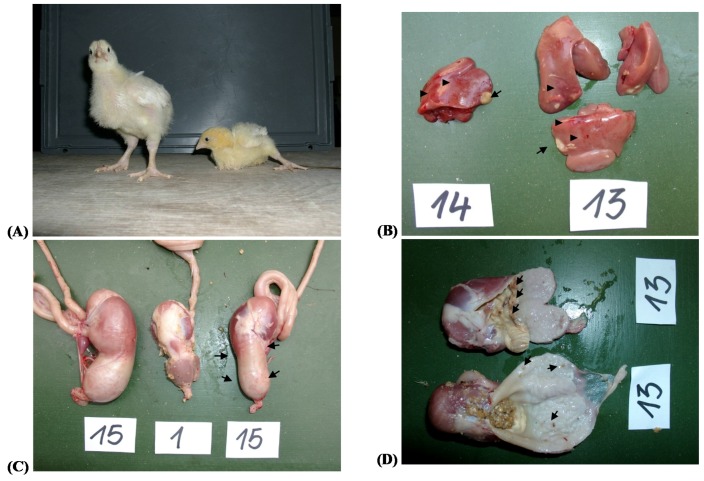
Clinical signs and pathological findings of ergot poisoning (30 g ergot/kg diet corresponding to 5.66–6.76 mg total ergot alkaloids/kg diet; groups 13–16): (**A**) broilers were partly unable to stand and displayed uncoordinated movements (left, control broiler of group 1; right, affected broiler of group 13); (**B**) livers showed multifocal hemorrhages (arrowheads) and necrotic lesions (arrows); (**C**) dilatation of the proventriculus paralleled by an apparent disappearing of the isthmus between proventriculus and gizzard (arrows); and (**D**) multifocal hemorrhages and necrotic lesions of proventricular mucosa (arrows).

The most obvious clinical sign of the presence of EA in the diets was a decrease in voluntary feed intake observed in groups fed diets containing 1 g ergot/kg or more with the effects being most pronounced at the highest ergot inclusion level (30 g/kg diet) (for a detailed evaluation see Section 2.3). Animals of groups fed diets with the highest ergot level of 30 g/kg (13–16) were partly unable to stand and displayed uncoordinated movements (Figure 1). Other clinical signs indicative for EA intoxication, such as discoloration, necrosis or gangrenous necrosis of the *Appendices integumenti* were not observed.

Total losses (mortality plus culling of moribund animals; expressed as percentage of the initial number of animals in the respective groups) until Day 14 of age amounted to 10.0%, 1.3%, 8.8% and 13.8% in control groups (1–4), groups fed diets containing 1 g ergot/kg diet (5–8), 10 g ergot/kg diet (9–12) and 30 g ergot/kg diet (13–16), respectively. Thus, neither EA exposure nor dietary fat content or NSP-enzyme supplementation could be identified as affecting total losses. 

Due to the pronounced clinical signs observed in groups 13–16 (30 g ergot/kg diet), it was decided for these birds to terminate the experiment after 14 days with a final necropsy of all animals of these groups. The most obvious organ pathology included a dilatation of the proventriculus which was associated with an apparent disappearing of the isthmus between proventriculus and gizzard (Figure 1) in 12.9% of all broilers of groups 13–16. This proventricular megalia was partly paralleled by multifocal bleedings of gastric mucosa, especially of the isthmus region (Figure 1). Such alterations were not observed in groups 1–4 (unexposed control birds). Further lesions were noticed for the duodenum where 4.6 and 6.3% of the broilers of group 13–16 and 1–4 were affected by a mild duodenitis. Five percent of the livers of groups 13–16 were affected by multifocal hemorrhages and necrotic lesions (Figure 1) while such hepatic pathologies were not observed in the control birds (groups 1–4). 

For groups that terminated the experiment regularly at Day 35 of age (groups 1–12), such organ lesions were not observed. 

### 2.3. Growth Performance

#### 2.3.1. Days 1 to 14 (Groups 1 to 16)

Feed intake decreased significantly after feeding the diets containing the highest ergot content of 30 g/kg diet while lower ergot proportions up to 10 g/kg diet failed to induce a decrease in feed intake (Table 4). Interestingly, there was a trend for an interaction between ergot and fat proportion (*p* = 0.053) which was due to the lower feed intake of the group fed the ergot-free diet without NSP-enzyme and containing 10% of soybean oil compared to groups fed diets containing 1 and 10 g ergot/kg. NSP-enzyme addition partially compensated this negative effect whereby a feed intake level similar to groups fed diets containing 1 and 10 g ergot/kg was reached. Live weight (LW) gain displayed a similar picture as described for feed intake (Table 4) with the addition that the described interactions between ergot and fat level, ergot level and NSP enzyme addition and fat level and NSP enzyme addition reached significance. Feed to gain ratio was significantly decreased at the higher dietary fat content and increased at the highest ergot level of 30 g/kg. Fat effects occurred at each ergot level in a similar direction while NSP-enzyme effects were not present. These effect relationships are substantiated by lack of any significant interactions. AME_N_ (apparent metabolizable energy, corrected for a zero N-retention)-intake closely mirrored the effect relationships described for feed intake with the exception that the NSP-enzyme effect reached significance. Similar to the feed to gain ratio the AME_N_ to gain ratio was characterized by similar significance relationships, i.e., a significantly lower ratio when the diets contained a higher fat content and a sharp increase of this ratio when diets with the highest ergot levels were fed. EA exposure clearly increased with dietary ergot level while the main effects of dietary fat level and NSP-enzyme addition were biased by significant interactions between all three fixed main factors.

Enzyme addition caused an increase in EA exposure at the low dietary fat level and at ergot levels of 1 and 10 g/kg diet, while at the highest ergot content in combination with the higher dietary fat content a decrease in exposure was noticed when compared to the corresponding counterpart.

#### 2.3.2. Days 1 to 35 of Age (Groups 1–12)

Feed intake significantly decreased with increasing dietary ergot contents in diets supplemented with the NSP-hydrolyzing enzyme preparation whereby the drop was more pronounced after feeding diets containing 1 g ergot/kg compared to 10 g ergot/kg (Table 5). The decrease was completely compensated when no NSP-hydrolyzing enzymes were added at dietary ergot levels of 1 g/kg irrespective of dietary fat content. However, at 10 g ergot/kg, the enzyme supplementation failed to exert any effect (*p*_ergot_ _× enzyme_ < 0.001). Moreover, absence of added enzymes resulted even in an increase in feed intake when the diet contained the lower fat and ergot level (*p*_ergot_ _× fat_ = 0.007). AME_N_ intake closely mirrored these dose and significance relationships. For live weight gain, the significant interaction between dietary ergot proportion and NSP-enzyme addition resulted from the variable, but always positive enzyme effects which appeared to be independent of dietary ergot proportion. The feed to gain ratio was markedly lower after enzyme addition, but only at an ergot content of 1 g/kg while no differences occurred when the diets contained no ergot or 10 g ergot/kg albeit at a comparably lower level for the latter which explained the significant interaction between ergot level and NSP enzyme supplementation. In addition, an interaction between dietary ergot and fat level was observed due to higher differences in feed to gain ratio between low and high fat diets at corresponding lower dietary ergot proportions compared to higher ones. AME_N_ intake to gain ratio displayed similar significance relationships excepting that the interactions between dietary ergot and fat levels failed to reach significance. EA exposure increased significantly with dietary ergot proportions whereby the increase was more pronounced in NSP enzyme supplemented broilers but solely at the lower dietary fat level resulting in significant interactions among all three fixed main factors. 

### 2.4. Clinical-Chemical Traits 

#### 2.4.1. Day 14: Groups 1–4 and 13–16

ASAT activity was significantly increased due to ergot presence in the diets except at the low dietary fat level causing a significant interaction between dietary ergot and fat level (Table 6). ALAT activity was consistently elevated after feeding the ergot containing diets irrespective of fat content and NSP-enzyme supplementation. GLDH activity was also increased due to ergot feeding but was additionally modified by NSP-enzyme supplementation and dietary fat level as it increased due to NSP enzyme supplementation after feeding the high fat diet while decreased when diets were not supplemented with the enzyme. As these effects occurred more pronounced when the ergot containing diets were fed, significant interactions between NSP-enzyme supplementation and dietary ergot level, and a tendency interaction between dietary fat level and NSP-enzyme supplementation were detected. GGT activity was significantly and consistently enhanced at the higher dietary fat level when the diet contained ergot while opposite effects were noticed for the ergot-free control diets where GGT activity increased with dietary fat level without NSP-enzymes, but decreased with addition of the enzymes. Albumin concentration significantly increased at higher dietary fat levels when diets were not supplemented with NSP-enzyme while enzyme addition resulted in an unaltered or even slightly decreased albumin concentration causing the significant interaction between NSP-enzyme addition and dietary fat level.

Total bilirubin concentrations were significantly higher in diets containing ergot and significantly reduced after enzyme supplementation. NDV antibody titers were significantly elevated in unsupplemented diets when ergot was present in feed while titers behaved similarly after feeding NSP-enzyme supplemented diets whereby the effect appeared to be more pronounced at higher dietary fat levels.

#### 2.4.2. Day 35: Groups 1–4 and 9–12

ASAT activity was significantly enhanced at the higher dietary fat level when no enzymes were added and the diet did not contain ergot while a decrease was noticed when ergot was present under these conditions (Table 7). In contrast, when enzymes were added to the diets a consistent decrease in ASAT activity was noticed at higher dietary fat proportions irrespective of ergot presence in the diets (Table 7) giving rise to the significant interactions between ergot presence and fat level, and between NSP-enzyme addition and dietary fat proportion. ALAT activity was significantly elevated at the lower dietary fat levels when the diets were free of ergot and decreased to the same constant and treatment-independent levels as measured for the groups fed ergot resulting in the significant interactions between ergot and fat content. GLDH activity was generally enhanced at higher dietary fat levels when diets remained unsupplemented by NSP enzymes. Adding the enzymes resulted in only small differences between the treatments and caused the significant interaction between dietary fat level and NSP enzyme supplementation. GGT activity significantly increased at higher dietary fat levels after feeding the ergot-free diets regardless of NSP-enzyme supplementation. However, the presence of ergot in the diets caused a drop in GGT activity at higher dietary fat levels when the diet was supplemented by NSP enzymes while no change was observed when these enzymes were supplemented. Albumin concentrations decreased consistently at higher dietary fat levels in ergot fed broilers whereby this effect occurred at a lower level when the diets were supplemented by the NSP enzymes. Similar relationships were detected when diets were free of ergot excepting when enzymes were added; here, the higher dietary fat proportion did not result in a decrease in albumin concentration. Total bilirubin concentration was significantly lower after feeding the high-fat diets. NDV titers were not significantly affected by treatments although it appeared that NSP-enzyme supplementation tended to increase the titers while higher dietary fat contents slightly decreased them. 

### 2.5. Organ Weights

Organ weights recorded after 14 days of ergot exposure relate to a dietary ergot content of 30 g/kg diet (groups 13–16) while those obtained after 35 days of exposure correspond to an ergot contamination level of 10 g/kg diet (groups 9–12).

#### 2.5.1. Day 14: Groups 1–4 and 13–16

Relative heart weights tended to be increased after feeding the ergot-contaminated diets for 14 days; especially in combination with diets not supplemented with NSP-enzymes (Table 8). Spleen weights were slightly increased due to feeding the ergot-contaminated diets. Feeding of ergot-contaminated diets resulted in lower relative weights of the *Bursa cloacalis* when compared to their control counterparts. Moreover, higher dietary fat contents induced an increased weight of this organ for ergot-exposed broilers while the opposite was observed in ergot-free fed groups resulting in a tendency interaction between ergot contamination and fat content of the diets. Relative liver weight was significantly enhanced due to feeding the ergot-contaminated diets while higher fat contents of the diets additionally decreased the liver weight significantly. 

#### 2.5.2. Day 35: Groups 1–4 and 9–12 

In addition to the relative weights of heart, spleen, bursa cloacalis and liver those of the duodenum, pancreas and proventriculus plus gizzard were recorded (Table 8). Ergot contamination of the diet tended to decrease the relative heart weights. The relative spleen weights were significantly increased after feeding the high-fat diets, whereas neither ergot contamination nor NSP-enzyme supplementation exerted a modifying effect. Relative weights of the bursa cloacalis were significantly increased at higher dietary fat proportions whereby this effect occurred at a lower level for ergot-containing diets after NSP-enzyme supplementation giving rise to a tendency interaction between ergot contamination and NSP-enzyme supplementation. Relative liver weights decreased at higher dietary fat contents in the absence of NSP-enzymes, an effect which occurred for the ergot-free diets at a higher level, while no treatment effect was detected when the enzymes were added to the diets which resulted in tendency interactions between NSP-enzyme supplementation and ergot contamination. The relative weight of the duodenum was significantly lower after feeding the ergot-containing diets but was generally higher and additionally influenced by the dietary fat content after feeding the diets not supplemented with NSP-enzymes.

Collectively, these effects resulted in significant interactions between ergot contamination, dietary fat content and NSP-enzyme addition. Relative weights of stomach plus proventriculus increased at higher fat contents when the diets were contaminated by ergot whilst the opposite was observed after feeding the ergot-free diets. These effects were independent of NSP-enzyme supplementation and resulted in significant interactions between dietary ergot contamination and fat content. 

### 2.6. Alkaloid Residues

#### Day 35: Groups 1–4 and 9–12

The concentrations of individual and of total EAs in blood, bile, liver and breast meat (without skin) were lower than the LOQ of 5 ng/g for all samples.

### 2.7. Apparent Nutrient Retention

Apparent fat retention was significantly higher in groups fed the high-fat diets compared to their low-fat counterparts while ergot contamination was without influence (Table 9). In contrast, nitrogen retention was significantly increased in ergot-fed birds whereby this effect appeared to be most pronounced at the highest dietary inclusion level of 30 g ergot/kg diet.

### 2.8. Viscosity of Intestinal Chyme 

**Figure 2:**
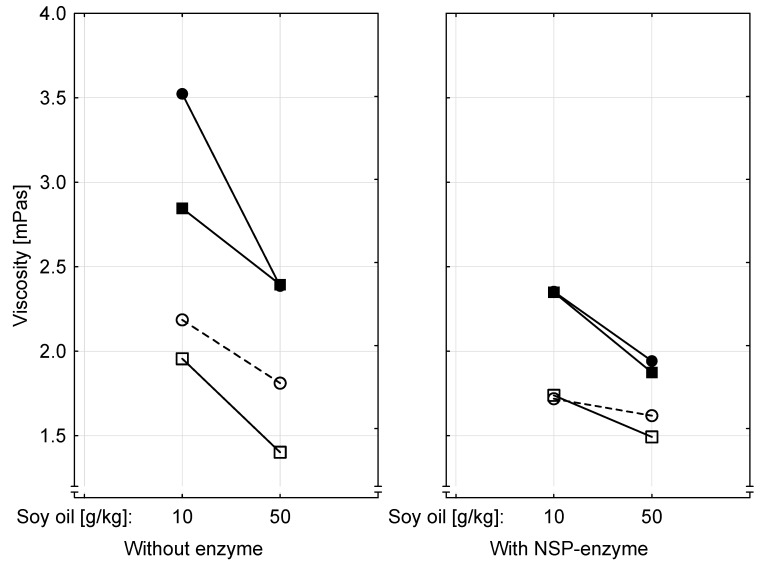
Interaction plot for the effects of supplementing the diets with a non-starch-polysaccharide (NSP) hydrolyzing enzyme preparation (ZY68, Lohmann Animal Health GmbH & Co. KG, Cuxhaven, Germany; declared activity: Endo-1,4-β-Xylanase (EC 3.2.1.8) 1000 FXU/g), of soy oil inclusion rate (10 or 50 g/kg diet), of absence or presence of ergot and of intestinal segment (---●--- , control-ileum; --○--, control-jejunum; ---■--- , 10 g ergot/kg diet-ileum; --□--, 10 g ergot/kg diet-jejunum) on viscosity of chyme; *p*-values for significant fixed main and interactive effects: *p*_intestinal segment_ < 0.001, *p*_enzyme_ < 0.001; *p*_ergot_ < 0.001, *p*_soy oil_ < 0.001, *p*_intestinal segment × enzyme_ = 0.005, *p*_soy oil × enzyme_ = 0.043; pooled standard error of means = 0.2 mPa·s. Three pooled samples were prepared each from jejunal and ileal chyme of 10 broilers per group.

Generally, the effects of dietary fat content and of ergot contamination on viscosity of jejunal and ileal chyme were more pronounced in diets not supplemented with the NSP-enzymes (Figure 2). Addition of these enzymes resulted in smaller differences between viscosity measured in jejunal and ileal chyme, between low- and high-fat diets and between ergot-free and ergot-containing diets. These relationships explain the significant interactions between intestinal segment and NSP-enzyme supplementation and between dietary fat level and NSP-enzyme addition. 

### 2.9. Deduction of LOAEL and NOAEL

**Figure 3:**
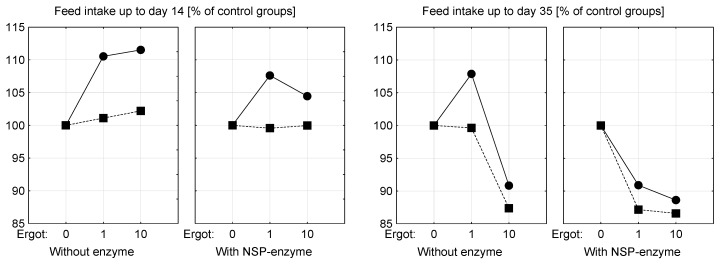
Feed intake of ergot exposed groups (1, 10g ergot/kg diet) relative to the control groups (%) fed diets without ergot (0g ergot/kg diet) up to: Day 14 (**left**); and Day 35 (**right**) (---●---, 10 g soy oil/kg diet; ---■---, 50 g soy oil/kg diet).

Feed intake was proven to respond most sensitive to the EA presence in the diets. This sensitivity appeared to be time-dependent which becomes obvious when feed intake of broilers, exposed to ergot containing diets either for 14 days or 35 days, is plotted relative to the corresponding control groups (Figure 3). Here, the LOAEL corresponded to a total dietary EA content of 5.7 mg/kg until Day 14 of age, while a lower LOAEL of 2.03 mg/kg was identified when birds were exposed for a period of 35 days. Consequently, NOAEL corresponded to an EA content of 2.49 mg/kg until Day 14 of age, while 1.94 mg/kg applied until Day 35 of age.

### 2.10. Risk Assessment 

**Figure 4:**
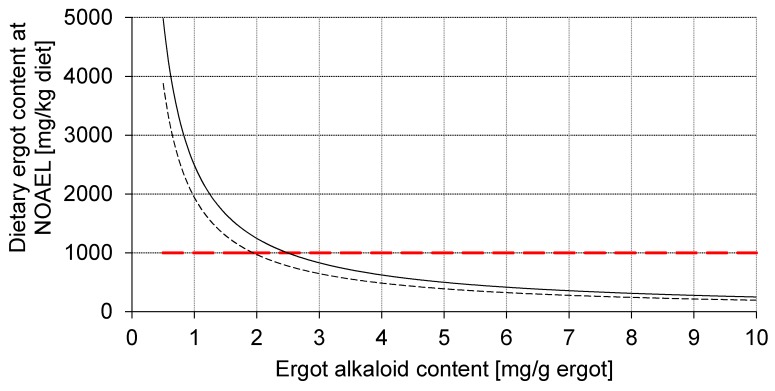
Dietary ergot content depending on ergot alkaloid (EA) content of ergot. The acceptable range of dietary ergot content in combination with its EA concentration is located left of the curves indicating the NOAEL of 2.49 mg EA/kg diet (up to 14 days of age, ────) and 1.94 mg EA/kg diet (up to 35 days of age, - - - - - - -). The red horizontal dashed line indicates the actual upper limit of 1000 mg ergot per kg unground cereals (Directive 2002/32/EC).

Since current feed safety regulations just specify an upper limit of 1000 mg ergot (*C. purpurea*) per kg unground cereal grains (Directive 2002/32/EC) which does not consider the variation in EA content of ergot as compiled by EFSA, it is necessary to relate the deduced LOAL and NOAEL to the expectable EA variation in ergot (Figure 4).

The NOAELs of 2.49 mg EA/kg diet (up to 14 days of age) and 1.94 mg EA/kg diet (up to 35 days of age) are plotted depending on EA content of ergot and the resulting dietary ergot content at NOAEL. Therefore, the area left to the corresponding curves represents the safe range of combinations of EA of ergot which does not exceed NOAEL. The red horizontal dashed line is additionally plotted as an orientation relative to the current feed safety regulations of 1000 mg ergot per kg unground cereals. It becomes clear that NOAEL is exceeded when EA content of ergot is higher than 1.9 mg/g (exposure up to 35 days of age) and 2.5 mg/g (exposure up to 14 days of age) when a dietary ergot content of 1000 mg/kg is assumed as upper limit.

## 3. Discussion

EAs are the compounds etiologically responsible for the classical signs of ergotism in humans and animals [1,4,7] associated with the presence of ergot in food and feed. Toxicologically, EAs potentially interact with serotoninergic, dopaminergic and adrenergic receptors depending on their specific chemical structures which mimic those of biogenic amines such as noradrenaline, dopamine (also known as prolactin inhibiting factor; prolactin inhibiting hormone) and serotonin [4].

It has been reviewed that acute doses of approximately 2–3 mg of ergotamine, ergometrine and ergotoxine per kg LW caused gangrenous ergotism in cocks [8]. In particular, clinical symptoms included a cyanosis of the comb and wattles which was followed by gangrene within 1 to 1.5 h after subcutaneous, oral or intramuscular administration. Other clinical signs were tachypnea, ataxia, salivation, defecation and drowsiness. In the present experiment the highest EA concentrations of 5.7–6.8 mg/kg diet analyzed in the diets containing 30 g ergot/kg corresponded to a mean EA exposure of 0.84–0.96 mg/kg LW within the 1st 14 days of the experiment. This exposure level was approximately 2–3 fold lower than the doses reported to cause gangrenous ergotism. The clinical signs observed in the present sub-chronical exposure scenario were dominated by ataxia, inability to stand and walk together with a pronounced decrease in voluntary feed intake and a consecutive depression in live weight development instead. Moreover, probably not only the lower exposure might have caused the differences in clinical signs between the earlier studies and the present experiment but also the fact that we used a mixture of EAs composed mainly of ergotamine (25%) and ergocristine (22%) besides much lower proportions of ergometrine, ergocryptine, ergosine and the corresponding -inine isomers. It has been suggested that the diversity of the biological effects of EAs results from their chemical diversity making it difficult to predict the net effect of an EA mixture on a clinical and pathological level due to dose and proportional-associated interactive effects. The latter are based on the fact that EAs interfere at more than one type of specific receptor site, that affinity and efficacy vary from alkaloid to alkaloid and that the population of receptor sites to which EAs have access varies from organ to organ [9]. Due to this complexity and the associated impossibility to assign observed toxic effects to individual EAs or their proportions to each other it seems to be an acceptable approach to discuss the results based on total EAs. Total EAs were demonstrated to be higher correlated with the toxic effects of ergot than the weight ergot proportion in piglet feed [10] supporting the view of evaluating toxic effects of ergot based on total alkaloids as a first approach. 

Clinical signs were strongly associated with dietary EA content while feed intake, live weight development and the corresponding live weight gain were further modified by dietary fat content and NSP-enzyme addition in an interactive manner. A higher dietary fat content generally improved live weight gain at all ergot levels but clearly failed to compensate the drop induced by the highest ergot inclusion level of 30 g/kg diet. As feed intake was not influenced to a similar extent the resulting feed to gain ratio as a measure of feed conversion was improved after feeding the fat enriched diets. This improved feed utilization can be explained by the markedly increased apparent fat retention of the fat enriched diets. Since only traces of lipids are renally excreted and consequently were collected with the excreta for analysis of fat, the apparent fat retention can to a large extent be regarded as fat absorption. The improved fat absorption associated with dietary fat did obviously not increase EA toxicity as ergot effects on live weight gain and feed to gain ratio were not aggravated by higher dietary fat contents. Beside the effects of dietary fat level on feed to gain ratio the ergot level markedly increased feed to gain ratio when broilers were exposed to diets containing 30 g ergot/kg for up to 14 days irrespective of dietary fat content and NSP-enzyme supplementation. At this exposure scenario, the drop in live weight gain was more pronounced than that for feed intake hinting at a decreased nutrient utilization. Interestingly, the nitrogen retention increased significantly at the same time suggesting that utilization of nitrogen was not compromised. When comparing these results from the balance experiment with the feed to gain ratio observed in the feeding trial it needs to be considered that broilers were fed restrictively in the balance experiment while birds of the growth trial had ad libitum access to feed. Moreover, although nitrogen retention comprises a substantial part of live weight gain, other nutrients such as fat, ash and also water contribute to it. Therefore, the apparently discrepancy between feed to gain ratio and nitrogen retention might be due to differences in feed intake level dependent differences in nutrient utilization and/or ergot effects on the retention of nutrients others than nitrogen. 

Addition of NSP-hydrolyzing enzymes to the diets differently modified the effects both of increasing dietary ergot and fat contents. These enzymes are added to commercial broiler diets on a routine basis to improve nutrient digestion and utilization as mediated by a decreased viscosity of intestinal chyme caused by soluble NSP. Such altered physico-chemical chyme conditions might have a number of physiological and nutritional consequences such as accelerated transit time of feed through the digestive tract and improved nutrient digestion and absorption which might also be relevant for EA toxicity. Interestingly, not only NSP-enzyme addition decreased the viscosity particularly of jejunal chyme but also higher fat contents and ergot further reduced viscosity. While the effects of NSP-enzyme addition and of dietary fat are well documented in the literature (e.g., [5] the ergot related chyme modification has not been reported so far. Based on the observation that especially dietary unsaturated fatty acids decrease intestinal viscosity [5], the here observed ergot-related viscosity-lowering effect might be due to the high fat content of ergot (~31%) together with a high proportion of unsaturated fatty acids such as oleic, linoleic and ricinoleic acid. However, other ergot compounds, and especially EAs, might also have contributed to this effect. Structural similarity of EAs with serotonin gives also rise to interactions at the enteric serotonin receptor level with consequences for the gastro-intestinal motility and secretions. Serotonin is known as gastrointestinal signaling compound and neurotransmitter produced by enterochromaffin cells and myenteric interneurons [11,12]. On the other hand, most EAs were shown to antagonize serotonin effects in a non-competitive and irreversible fashion in stomach [13]. Moreover, interactions between serotonin and EAs were reported for esophagus, duodenum, jejunum, ileum, colon, caecum and rectum preparations of various species, including fowl [11,12]. These findings might suggest that contact of ingested EAs with serotonin receptors of the enteric nervous system might have influenced the gastro-intestinal motility, including that of the proventriculus. An inhibited stimulation of serotonin receptors of proventriculus or differently dysregulated motility could have induced a loss of tone of smooth muscles and a consecutive dilatation. 

At the level of feed intake and live weight gain the NSP-enzyme addition resulted in an improvement which, however, got smaller with increasing dietary ergot contents. This effect appeared to be more pronounced when diets with the lower fat content were fed. 

Relative liver weight, gross pathological findings and clinical-chemical traits (ASAT, GLDH, ALAT, total bilirubin) clearly hinted at hepatotoxic effects of EAs when diets contained 5.7–6.8 mg/kg while lower concentrations failed to induce such pronounced effects. Similar findings were reported for laying hens and Pekin ducks fed diets containing 13.7 mg and 11.4 mg/kg for 20 and 7 weeks, respectively [6,14]. Liver weights were significantly increased by 7% and 10% compared to the control groups in hens and ducks, respectively, while an average increase of 17% was noticed in the present experiment with broilers. 

Enlarged livers have been associated with increased hepatocellular glycogen storage in rats fed diets containing up to 250 mg ergometrine maleate/kg for four weeks [15], or 20–500 mg ergocryptine/kg for 28–32 days [16,17]. As no histopathology was performed in the present experiment the nature of the hepatomegalia needs to be examined further.

GLDH responded most sensitively amongst the clinical-chemical traits to EAs in the present experiment with a mean increase by 98% compared to the controls. In contrast, in hens total bilirubin (+56%) appeared to indicate the toxicity of EAs more sensitively and in ducks the GGT impressed by an increase of 92% due to ergot feeding. However, the other liver lesion more or less indicating clinical-chemical traits were either significantly or numerically increased in hens, broilers and ducks. 

Interestingly, GLDH activity was not only generally increased in ergot-exposed broilers but was additionally enhanced when the ergot-contaminated diets were supplemented with the NSP-hydrolyzing enzymes giving rise to the significant interaction between NSP-enzyme supplementation and dietary ergot contamination. In contrast, the enzyme supplementation decreased GGT activity and total bilirubin concentration. While the enzyme supplementation-associated GLDH activity increase would hint at an increased loss of hepatocyte integrity, the decreased GGT activity and total bilirubin concentration could indicate less pronounced cholestatic effects. Nonetheless, the NSP-enzyme addition appeared to influence the liver systematically with the effect being more pronounced in the presence of high dietary ergot levels of 30 g/kg. The nature of these effects remains largely speculative but might be the indirect consequence of NSP-hydrolyzing enzyme effects on physico-chemical chyme characteristics and their consequences for nutrient digestion and absorption as discussed above. 

Pathological findings as observed in the present experiment might have functional consequences as reported for piglets. It could be demonstrated that the liver function or more precisely, the activity of the cytochrome P4501A2 is significantly reduced when dietary EA concentration exceeds 17 and 21 mg/kg diet for male and female piglets, respectively [10,18,19]. These critical EA levels were much higher than those affecting feed intake and live weight gain adversely. Considering blood albumin concentration as a liver function indicator it might be deduced that liver function was not compromised in the present experiment. It might be concluded that albumin is not a fully reliable liver function indicator as experiments with hens [6] and ducks [14] showed that liver pathologies were associated with a significant increase in blood albumin concentration. Therefore, no final conclusion can be drawn on the functional consequences of hepatotoxicity of EAs in broilers, hens and ducks. 

Effects of EAs on immune cells are discussed to result from their interactions with membrane receptors [20]. The EAs elymoclavine and DH-lysergol were demonstrated to modulate the natural killer (NK) cell-mediated cytotoxicity of human resting and peripheral blood mononuclear cells (PBMC) [20]. While elymoclavine stimulated NK cell mediated cytotoxicity the alkaloid DH-lysergol exerted only marginal effects suggesting that the pattern of EAs present in naturally contaminated feed might be decisive for the net outcome of immune responses. Effects of EAs on the immune system of the chicken have hardly been studied so far. In a study with laying hens the antibody response to NDV vaccination was found to be stimulated in the presence of EAs. Although we also detected stimulatory EA effects on NDV titers in the present experiment with broilers this effect only occurred in the absence of the NSP enzyme and at 14 days of age. Interactions between NSP enzyme supplementation and wheat batch origin on NDV titers were also reported for broilers [21]. In this experiment, the NSP enzyme preparation resulted in a stimulated antibody response only when it was added to the wheat-based control diet while the NSP enzymes were ineffective in combination with a Fusarium toxin contaminated wheat batch. The reasons for these interactions and those observed in the present experiment hint at an involvement of physic-chemical chyme conditions on antibody response since NSP hydrolyzing enzymes act at this site. 

No residues of free EAs were detectable in blood, bile, liver and breast meat (without skin) with the used HPLC-method and the indicated LOQs. The analytical method detected only free -in and -inine forms of EA. Degraded or otherwise modified EA-forms, including lysergic acid and its derivatives, hydroxylated and de-methylated forms [22,23] could not be found. In contrast to the present experiment, ergometrine and ergometrinine were detected in bile of laying hens [6] and Pekin ducks [14] while residues in edible tissues, including eggs, were not detected either. In the present experiment the residue analyses based on samples collected from broilers of treatment groups 9-12 which were fed diets with total EA concentrations between 1.88 and 2.03 mg/kg. However, hens and ducks received diets with higher EA concentrations (13.03/14.56 and 6.95 mg/kg, respectively), which might explain that detection of EAs failed in the present experiment. In ruminants, necrosis of adipose tissues was observed after grazing cattle on alkaloid-containing pastures. This pathology was suggested to be related to the presence of EAs in adipose tissues [24]. Whether EAs cause fat necrosis and accumulate in adipose tissues of broilers such as subcutaneous and abdominal adipose tissue was not addressed in the present and should be examined in future experiments. 

## 4. Conclusions

Feed intake was identified as the most sensitive endpoint suitable for deducing both LOAEL and NOAEL. Chronic exposure up to 35 days from hatching appeared to depress feed intake at lower dietary EA contents (LOAEL = 5.7 mg/kg diet) compared to a sub-chronical exposure up to 14 days (LOAEL = 2.07 mg/kg diet). The corresponding NOAELs were identified at 2.49 mg/kg diet until Day 14 of age, and at 1.94 mg/kg diet until Day 35 of age. Hepatotoxicity was a common feature of EA and dilatation of proventriculus could hint at local interactions between EAs and serotonin receptors. 

NSP-hydrolyzing enzyme addition and dietary fat level have been identified as factors influencing ergot toxicity. Both factors did not aggravate toxic effects of EAs but rather counterbalanced adverse effects on performance up to an EA level of approximately 2 mg/kg diet (10 g ergot/kg diet). 

The risk assessment made clear why broilers are not sufficiently protected by current feed safety regulations, especially at higher EA contents of ergot.

## 5. Materials and Methods 

The experiments were conducted according to the European Community regulations concerning the protection of experimental animals and the guidelines of the Regional Council of Braunschweig, Lower Saxony, Germany (File number 509b.42502/2-6).

### 5.1. Experimental Design

The study was planned according to a complete 4 by 2 by 2 three-factorial design. Four levels of ergot (0, 1, 10 and 30 g ergot/kg diet) were combined with 2 different fat concentrations (10 and 50 g/kg diet) which were prepared without or with an NSP-hydrolyzing enzyme preparation (0 and 2 g/kg diet, ZY68, Lohmann Animal Health GmbH & Co. KG, Cuxhaven, Germany; declared activity: Endo-1,4-β-Xylanase [EC 3.2.1.8] 1000 FXU/g) (Table 10). 

Ergoty rye containing 60% ergot was used as ergot source and was included in the diets at 0, 1.7, 17 and 50 g/kg diet to reach the target ergot levels of 0, 1, 10 and 30 g ergot/kg diet, respectively (Table 3). All diets were adjusted to contain a total rye content of 50 g/kg. Furthermore, all diets were designed to be iso-energetic by incorporating either maize starch or cellulose to balance the different fat contents of 10 and 50 g/kg diet. By this approach the diets were also kept at a similar crude protein content.

### 5.2. Growth Experiment

A total of 1280 day-old male broilers of the strain Lohmann Meat (“WIESENHOF Geflügel-Kontor GmbH” company, Visbeck, Germany) were used in the experiment. They were kept in a total of 160 cages in an experimental unit enabling a controlled lighting and temperature regimen according to the recommendations of the breeder. 

Broilers were randomly assigned to the 16 treatments which were replicated 10 times. Each replicate consisted of 8 birds resulting in a total of 80 chicks per treatment. The average initial live weight was similar for all groups and amounted to 47.6 ± 1.4 g. Feed and water were offered for ad libitum consumption. Chicks were vaccinated via drinking water with a live Newcastle disease virus (NDV) vaccine (LaSota, 109EID50). Broilers were weighed weekly and the corresponding consumed feed amount was determined as the difference between the amounts of feed offered and weighed back. 

The described procedures were applied to all 16 groups until Day 14 of age. Due to serious health problems the experiment was terminated for groups 13 to 16 (highest EA exposure; for further details see paragraph “Clinical signs and organ pathology”) with the necropsy of all broilers of these 4 groups. As organ weights and gross-macroscopical organ pathology should have been compared to unexposed broilers at this time point, additionally all animals of always 1 cage of group 1 to 4 (*n* = 8/group) were necropsied. The remaining groups 1 to 12 terminated the experiment after 35 days as planned. 

After the final weighing of groups 1 to 12, 10 broilers of treatments 1–4 and 9–12 were slaughtered by cutting the neck vessels after manual stunning. Mixed trunk blood was collected from the neck vessels for determination of antibody titers and EA residue analysis. Jejunum (from the entry of the main bile and pancreatic ducts to the Meckel’s diverticulum), ileum (from the Meckel’s diverticulum to the ileo-cecal junction), pancreas, liver, spleen, bursa cloacalis and heart were quickly dissected. Ingesta from the jejunum and ileum were collected in pre-cooled tubes, pooled for 3 samples per group and kept on ice before being frozen for later determination of viscosity. Bile was sampled by puncturing the gall bladder and pooled to 3 samples for EA residue analysis.

Weights of heart, spleen, liver and bursa cloacalis were recorded individually at days 14 and 35 of age, while pancreas, emptied duodenum and proventriculus and gizzard were weighed only at Day 35 of age. Breast meat and livers (without gall bladder) were pooled to 3 samples each and kept frozen before being further processed for EA residue analysis. 

### 5.3. Balance Experiment

A balance study with a total of 36 broilers of the same hatch as used in the growth experiment was performed during weeks 3 and 4 of age (mean initial live weight of 188.6 ± 13.4 g) according to the total collection method [25]. 

For capacity reasons only 6 diets were tested in the balance experiment and included 6 broilers of each group 1, 2, 9, 10, 13 and 14. Therefore, only the effects of increasing dietary ergot and fat proportions on apparent nutrient retention could be examined while NSP-enzyme supplementation could not be assessed. 

Broilers were placed into individual balance cages and adjusted to a daily feed amount of initially 50 g during adaptation period, and 60 g per animal during the excreta sampling period of the low-fat control diet during the first 6 days. Thereafter, experimental diets replaced 25%, 50%, 75% and 100% of the daily offered feed amount. Following to this adaptation, 100% of the test diets were fed during the next 8 days. Excreta were totally collected during the last 4 days from the plastic trays beneath the cages in the morning and in the afternoon, and were frozen at −20 °C between samplings. After finishing the collection period the excreta were freeze-dried and ground to pass through a 1 mm screen for chemical analyses.

### 5.4. Analyses

#### 5.4.1. Nutrients 

Diets were analyzed for dry matter (DM), nitrogen (N), crude ash (CA), crude fat (CL), crude fiber (CF), starch and sugar according to the official standard methods of the Association of German Agricultural Research and Investigation Institutions (VDLUFA) [26]. Freeze dried excreta collected during the balance experiment were analyzed for DM, N, CA and CF. DM, CL, CA, CF and N were determined in ergot for characterization of its crude nutrient composition. 

Fatty acids in ergot were analyzed by gas chromatography as described in detail elsewhere [27,28].

NSP concentrations in wheat, rye and ergot were determined according to the Uppsala method [29].

#### 5.4.2. Ergot Alkaloids

By an HPLC method [30] with slight modifications as described in detail earlier [31], EAs (ergometrine, ergocornine, ergotamine, α-ergocryptine, ergosine, ergocristine and their -inine isomers) in the ergot, diets, serum, bile, liver and breast meat (without skin) were analyzed. 

In brief, samples of approximately 5 g were extracted with dichloromethane/ethylacetate/methanol/25% ammonium hydroxide (50 + 25 + 5 + 1) overnight. After centrifugation, an aliquot was evaporated to dryness. The residue was cleaned-up using Extrelut^R^ columns (Merck, Darmstadt, Germany). Finally, the sample was filled up to a volume of 500 mL in the mobile layer of the HPLC [acetonitrile/water (1 + 1); with ammonium carbonate adjusted on pH 8.4] from which 20 mL were injected in the HPLC-system; consisting of an isocratic pumping system, operated at 44 °C, and fluorescence detector (325 nm excitation/418 nm emission wave length).

For each of the -ine and -inine EA forms the limit of quantification (LOQ) amounted to 5 ng/g at a sample size of 5 g for all specimens. The recovery rate varied between 50 and 139% depending on matrix and specific alkaloid, resulting in an average of 83%. Measured alkaloid concentrations were not adjusted for recovery. The standards of ergometrine, ergotamine, ergocristine, ergocornine and ergocryptine were commercially available for their identification (Sigma-Aldrich Chemie GmbH, Buchs, Switzerland). The same standards were also applied for the identification of their corresponding -inine isomers while ergosine and its isomers were determined through their retention time [32]. Taking into account their known instability the necessary standards were prepared freshly and stored in dark glassware in the refrigerator before analyzing a sample series. The sum of all identified alkaloids (-ine and -inine isomers) is referred to as “total alkaloids”.

#### 5.4.3. Viscosity of Intestinal Chyme and Clinical-Chemical Blood Characteristics

Viscosity of jejunal and ileal chyme was determined by using a Brookfield viscometer as described by Dusel, et al. [33].

Serum clinical-chemical parameters were determined using test-kits supplied by Merck, Darmstadt, Germany: glutamate dehydrogenase (GLDH, EC 1.4.1.3, Merck-1-Test^®^, 1.03373), gamma-glutamyltransferase (GGT, EC 2.3.2.2, Granutest^®^ 3, 1.12189.0001), aspartate aminotransferase (ASAT, EC 2.6.1.1, Granutest^®^ 3, 12150), alanine aminotransferase (ALAT, EC 2.6.1.2, Granutest^®^ 3, 1.12166.0001), total bilirubin (Merck-1-Test^®^, 1.03333.0001) and albumin (Granutest^®^ 1.14819.0001). Antibody titers to NDV in serum were ascertained by a hemagglutination-inhibition test (micro-method). 

### 5.5. Calculations and Statistics

Daily feed intake was calculated from the difference between the amount of feed offered for ad libitum consumption and the feed residuals weighed back divided by the number of broilers per cage and the corresponding period length (14 and 35 days, respectively). Daily live weight gain was determined as the differences between the individually body weights recorded at consecutive time points divided by the corresponding length of period (14 and 35 days, respectively). The corresponding feed to gain ratios were obtained by dividing daily feed intake by live weight gain. Daily EA exposure was calculated by multiplying the mean daily feed intake with the analyzed EA concentration of the corresponding diet divided by the mean body weight of the respective period (i.e., [body weight at beginning + body weight at the end of the period] divided by 2). 

Results were evaluated according to a complete 3-factorial design of analysis of variance (ANOVA) with dietary ergot level, fat concentration, NSP enzyme supplementation and the interactions between these main effects as fixed factors except for the balance experiment where the design was reduced to a 2-factorial design. 

Results are reported as means and pooled standard error of means (PSEM). Tukey test was used for post-hoc testing of differences between means in case of significance of the fixed factors. 

All statistics were performed using STATISTICA 12.0 (StatSoft, Inc. 2014, Tulsa, OK, USA).

## Figures and Tables

**Table 1:** Chemical composition of ergot.

Dry matter (g/kg)	923.1	Alkaloids [mg/kg] ^1^	
**Crude nutrients [g/kg] ^1^**		Total alkaloids ^2^	211.4
Crude ash	31.4	Key alkaloids ^3^	131.7
Crude protein	191.8	Ergometrine	5.2
Crude fat	306.7	Ergometrinine	2.2
Crude fibre	257.8	Ergotamine	52.3
**Fatty acid composition [g/100 g crude fat]**		Ergotaminine	12.0
Capric acid (C10:0)	0.04	Ergocornine	13.4
Lauric acid (C12:0)	1.76	Ergocorninine	7.6
Myristic acid (C14:0)	0.46	Ergocristine	47.2
Palmitic acid (C16:0)	29.20	Ergocristinine	15.0
Palmitoleic acid (C16:1)	3.49	Ergocryptine	13.6
Stearic acid (C18:0)	6.74	Ergocryptinine	15.2
Oleic acid (C18:1)	21.43	Ergosine	21.8
Linoleic acid (C18:2)	17.35	Ergosinine	5.9
Linolenic acid (C18:3)	0.73		
Arachidic acid (C20:0)	1.27		
Eicosenoic acid (C20:1)	0.25		
Eicosadienoic acid (C20:2)	0.07		
Behenic acid (C22:0)	0.36		
Erucic acid (C22:1)	0.17		
Ricinoleic acid (12-OH-C18:1)	16.50		
Lignoceric acid (C24:0)	0.18		

^1^ Based on a dry matter content of 880 g/kg; ^2^ Sum of ergometrine, ergotamine, ergocornine, ergocristine, ergocryptine, and ergosine and of their -inin-isomeres; ^3^ Sum of ergometrine, ergotamine, ergocornine, ergocristine, and ergocryptine.

**Table 2:** Composition of non-starch-polysaccharides (NSP) of wheat, rye and ergot.

	Wheat		Rye		Ergot	
	Soluble	Total	Soluble	Total	Soluble	Total
Individual sugars (% of total)						
l-rhamnose	0.7	0.3	0.3	0.3	0.5	0.1
d-fucose	0.7	0.1	0.3	0.2	0.0	0.0
l-arabinose	24.4	24.9	29.1	23.8	2.1	2.0
d-xylose	35.3	40.9	43.8	34.4	2.1	0.4
d-mannose	2.1	3.6	3.0	4.5	36.3	30.8
d-galactose	17.9	2.6	5.5	3.5	34.7	19.8
d-glucose	18.9	27.6	18.0	33.3	24.3	46.9
Sum (g/kg DM)	14.6	93.4	38.9	112.5	9.5	94.9

**Table 3:** Composition of experimental diets.

	Group															
	1	2	3	4	5	6	7	8	9	10	11	12	13	14	15	16
**Components (g/kg)**																
Wheat	366.1	366.1	364.1	364.1	365.08	365.08	363.08	363.08	355.9	355.9	353.9	353.9	336.1	336.1	334.1	334.1
Rye	50	50	50	50	49.32	49.32	49.32	49.32	43.2	43.2	43.2	43.2	30	30	30	30
Cellulose	0	60	0	60	0	60	0	60	0	60	0	60	0	60	0	60
Maize starch	125	25	125	25	125	25	125	25	125	25	125	25	125	25	125	25
Ergoty rye	0	0	0	0	1.7	1.7	1.7	1.7	17	17	17	17	50	50	50	50
Soybean meal	225	225	225	225	225	225	225	225	225	225	225	225	225	225	225	225
Soybeans	180	180	180	180	180	180	180	180	180	180	180	180	180	180	180	180
Soybean oil	10	50	10	50	10	50	10	50	10	50	10	50	10	50	10	50
Di-calcium phosphate	18.5	18.5	18.5	18.5	18.5	18.5	18.5	18.5	18.5	18.5	18.5	18.5	18.5	18.5	18.5	18.5
Calcium carbonate	10.4	10.4	10.4	10.4	10.4	10.4	10.4	10.4	10.4	10.4	10.4	10.4	10.4	10.4	10.4	10.4
Sodium chloride	2.5	2.5	2.5	2.5	2.5	2.5	2.5	2.5	2.5	2.5	2.5	2.5	2.5	2.5	2.5	2.5
dl-methionine	2.5	2.5	2.5	2.5	2.5	2.5	2.5	2.5	2.5	2.5	2.5	2.5	2.5	2.5	2.5	2.5
Premix ^1^	10	10	10	10	10	10	10	10	10	10	10	10	10	10	10	10
ZY68 ^2^	0	0	2	2	0	0	2	2	0	0	2	2	0	0	2	2
**Calculated composition (g/kg)**																
Crude protein	212.2	212.3	212.2	212.3	212.3	212.4	212.3	212.4	213.4	213.5	213.4	213.5	216	216.1	216	216.1
Crude fat	52.5	92.2	52.5	92.2	52.8	92.5	52.8	92.5	55.6	95.3	55.6	95.3	61.9	101.5	61.9	101.5
AME_N_^3^ (MJ/kg)	12.2	12.3	12.2	12.3	12.2	12.3	12.2	12.3	12.2	12.3	12.2	12.3	12.2	12.3	12.1	12.3
Lysine	12	12	12	12	12	12	12	12	12	12	12	12	12.2	12.2	12.2	12.2
Methionine + cysteine	9.2	9.2	9.2	9.2	9.2	9.2	9.2	9.2	9.2	9.2	9.2	9.2	9.3	9.3	9.3	9.3
Methionine	6.1	6.1	6.1	6.1	6.1	6.1	6.1	6.1	6.1	6.1	6.1	6.1	6.1	6.1	6.1	6.1
Threonine	5.2	5.2	5.2	5.2	5.2	5.2	5.2	5.2	5.2	5.2	5.2	5.2	5.1	5.1	5.1	5.1
Tryptophan	1.7	1.7	1.7	1.7	1.7	1.7	1.7	1.7	1.7	1.7	1.7	1.7	1.7	1.7	1.7	1.7
Calcium	9.6	9.6	9.6	9.6	9.6	9.6	9.6	9.6	9.6	9.6	9.6	9.6	9.7	9.6	9.7	9.6
Total phosphorus	7	7	7	7	7	7	7	7	7	7	7	7	7.1	7.1	7.1	7.1
Sodium	1.1	1.1	1.1	1.1	1.1	1.1	1.1	1.1	1.1	1.1	1.1	1.1	1.1	1.1	1.1	1.1
Total alkaloids (mg/kg)	0	0	0	0	0.22	0.22	0.22	0.22	2.15	2.15	2.15	2.15	6.34	6.34	6.34	6.34
**Analyzed composition (g/kg)**																
Dry matter	899.1	904.1	896.4	904.4	896.6	902.3	895.5	903.0	900.4	903.0	901.5	905.7	901.0	906.2	900.3	904.5
Crude protein	207.0	206.9	208.9	207.8	207.2	207.9	205.4	209.2	211.0	211.9	211.6	213.6	219.1	221.7	215.3	217.3
Crude fat	69.4	101.1	71.8	108.4	71.3	109.4	72.1	108.2	73.5	112.0	71.7	109.7	80.7	117.3	81.5	119.2
Crude fibre	31.3	70.7	28.6	68.2	30.0	68.9	30.5	66.3	29.9	71.9	30.4	71.5	34.1	73.1	36.2	74.7
Starch	376.4	304.1	387.0	297.6	362.0	283.7	371.5	287.2	369.8	278.3	363.6	283.7	356.8	269.0	358.4	274.2
Sugar	42.7	41.1	42.5	42.5	43.3	41.4	41.7	44.3	42.0	41.4	42.8	42.8	41.4	41.5	41.3	40.5
Crude Ash	61.1	60.3	60.7	60.0	60.2	61.3	61.0	60.2	61.7	61.9	61.3	62.3	58.4	62.3	62.8	61.7
AME_N_ (MJ/kg)	12.4	12.3	12.7	12.5	12.3	12.3	12.4	12.3	12.5	12.3	12.4	12.4	12.7	12.5	12.7	12.6
Total alkaloids (mg/kg)	0.13	0.25	0.14	0.08	0.25	0.25	0.46	0.28	1.88	1.94	2.49	2.03	5.76	6.76	5.66	5.85

^1^ Premix provided per kg of diet: 4.8 mg retinol, 100 µg cholecalciferol, 40 mg dl-α-tocopheryl acetate, 2.3 mg thiamine, 9.5 mg riboflavin, 15 mg calcium-d-pantothenate, 45 mg nicotinic acid, 6 mg pyridoxin-hydrochloride, 1.2 mg folic acid, 32 µg cyanocobalamin, 4.5 mg menadione, 50 µg biotin, 550 mg choline chloride, 80 mg Zn, 30 mg Fe, 100 mg Mn, 20 mg Cu, 0.4 mg Co, 1.2 mg J, 0.4 mg Se, 100 mg butylhydroxytoluol. ^2^ ZY68, Lohmann Animal Health GmbH & Co. KG, Cuxhaven, Germany; Declared activity: Endo-1,4-β-Xylanase (EC 3.2.1.8) 1000 FXU/g. ^3^ apparent metabolizable energy, corrected for a zero N-retention.

**Table 4:** Growth performance and ergot alkaloid (EA) exposure depending on dietary ergot, fat level and supplementation with a xylanase containing enzyme preparation up to 14 days of experiment (*n* = 10).

Group	Ergot (g/kg)	Soy Oil (g/kg)	Enzyme ^1^	Live Weight (LW) Gain (g/d)	Feed Intake (g/d)	Feed to Gain Ratio (g/g)	EA Exposure (µg/kg LW/d)	AME_N_-Intake (kJ/d)	AME_N_ to Gain Ratio (kJ/g)
1	0	10	−	21.9 ^d^	31.4 ^b^	1.434 ^ef^	20 ^a^	391 ^b^	17.8 ^ef^
2	0	50	−	26.4 ^f^	34.1 ^cd^	1.288 ^a^	37 ^ab^	419 ^c^	15.8 ^ab^
3	0	10	+	25.0 ^e^	33.0 ^bc^	1.326 ^ab^	21 ^a^	420 ^c^	16.9 ^cde^
4	0	50	+	27.2 ^h^	34.9 ^cd^	1.284 ^a^	12 ^a^	435 ^c^	16 ^abc^
5	1	10	−	25.6 ^ef^	34.8 ^cd^	1.358 ^c^	38 ^ab^	426 ^c^	16.7 ^bcd^
6	1	50	−	27.0 ^h^	34.4 ^cd^	1.274 ^a^	36 ^ab^	422 ^c^	15.6 ^a^
7	1	10	+	26.5 ^fg^	35.6 ^d^	1.346 ^bc^	70 ^b^	441 ^c^	16.7 ^bcd^
8	1	50	+	27.6 ^h^	34.8 ^cd^	1.258 ^a^	40 ^ab^	429 ^c^	15.5 ^a^
9	10	10	−	25.2 ^e^	35.1 ^cd^	1.392 ^de^	294 ^c^	439 ^c^	17.4 ^de^
10	10	50	−	26.7 ^gh^	34.8 ^cd^	1.305 ^a^	288 ^c^	429 ^c^	16.1 ^abc^
11	10	10	+	25.4 ^e^	34.5 ^cd^	1.36 ^cd^	382 ^d^	427 ^c^	16.8 ^bcde^
12	10	50	+	27.0 ^h^	34.9 ^cd^	1.294 ^a^	300 ^c^	432 ^c^	16 ^abc^
13	30	10	−	12.0 ^a^	19.4 ^a^	1.619 ^h^	850 ^e^	246 ^a^	20.5 ^h^
14	30	50	−	14.5 ^c^	21.4 ^a^	1.48 ^fg^	970 ^f^	267 ^a^	18.5 ^fg^
15	30	10	+	13.0 ^ab^	21.5 ^a^	1.655 ^h^	876 ^e^	272 ^a^	20.9 ^h^
16	30	50	+	13.6 ^bc^	20.5 ^a^	1.514 ^g^	841 ^e^	258 ^a^	19 ^g^
***p-values***									
Ergot				<0.001	<0.001	<0.001	<0.001	<0.001	<0.001
Fat				<0.001	0.130	<0.001	0.600	0.448	<0.001
Enzyme				0.003	0.151	0.329	0.882	0.045	0.722
Ergot × fat				0.011	0.053	0.409	<0.001	0.124	0.278
Ergot × enzyme				0.040	0.586	0.162	<0.001	0.224	0.284
Fat × enzyme				0.028	0.236	0.290	<0.001	0.265	0.251
Ergot × fat × enzyme				0.217	0.394	0.509	0.001	0.297	0.614
PSEM				0.5	0.7	0.029	13	9	0.4

^1^ “−” without and “+” with supplementation of ZY68, Lohmann Animal Health GmbH & Co. KG, Cuxhaven, Germany; Declared activity: Endo-1,4-β-Xylanase (EC 3.2.1.8) 1000 FXU/g, ^a–g^ values with no common superscripts are significantly different within columns (*p* < 0.05), PSEM = pooled standard error of means.

**Table 5:** Growth performance and ergot alkaloid (EA) exposure depending on dietary ergot, fat level and supplementation with a xylanase containing enzyme preparation up to 35 days of experiment (*n* = 9).

Group	Ergot (g/kg)	Soy Oil (g/kg)	Enzyme ^1^	Live Weight (LW) Gain (g/d)	Feed Intake (g/d)	Feed to Gain Ratio (g/g)	EA Exposure (µg/kg LW/d)	AME_N_-Intake (kJ/d)	AME_N_ to Gain Ratio (kJ/g)
1	0	10	−	54.1 ^a^	88.6 ^b^	1.64 ^cd^	12 ^b^	1102 ^b^	20.4 ^d^
2	0	50	−	57.2 ^bc^	91.8 ^bc^	1.604 ^cd^	22 ^c^	1128 ^bcd^	19.7 ^c^
3	0	10	+	56.9 ^b^	90.9 ^bc^	1.599 ^c^	12 ^b^	1156 ^cde^	20.3 ^d^
4	0	50	+	58 ^bc^	93.4 ^cd^	1.61 ^cd^	7 ^a^	1164 ^de^	20.1 ^cd^
5	1	10	−	58.1 ^bc^	95.6 ^d^	1.647 ^d^	22 ^c^	1173 ^e^	20.2 ^cd^
6	1	50	−	56.4 ^b^	91.4 ^bc^	1.622 ^cd^	22 ^c^	1120 ^bc^	19.9 ^cd^
7	1	10	+	59 ^c^	82.6 ^a^	1.401 ^ab^	35 ^d^	1024 ^a^	17.4 ^ab^
8	1	50	+	59.1 ^c^	81.4 ^a^	1.378 ^a^	21 ^c^	1004 ^a^	17 ^a^
9	10	10	−	56.6 ^b^	80.5 ^a^	1.423 ^b^	146 ^e^	1007 ^a^	17.8 ^b^
10	10	50	−	56.5 ^b^	80.2 ^a^	1.419 ^ab^	150 ^f^	987 ^a^	17.5 ^ab^
11	10	10	+	58.2 ^bc^	80.5 ^a^	1.384 ^ab^	188 ^h^	996 ^a^	17.1 ^a^
12	10	50	+	57.4 ^bc^	80.9 ^a^	1.41 ^ab^	156 ^g^	1001 ^a^	17.4 ^ab^
***p-values***									
Ergot				0.005	<0.001	<0.001	<0.001	<0.001	<0.001
Fat				0.420	0.940	0.369	<0.001	0.296	0.019
Enzyme				<0.001	<0.001	<0.001	<0.001	0.001	<0.001
Ergot × fat				0.005	0.007	0.298	<0.001	0.043	0.252
Ergot × enzyme				0.790	<0.001	<0.001	<0.001	<0.001	<0.001
Fat × enzyme				0.684	0.452	0.152	<0.001	0.440	0.139
Ergot × fat × enzyme				0.129	0.564	0.605	<0.001	0.461	0.410
PSEM				0.7	1.2	0.016	1	15	0.2

^1^ “−” without and “+” with supplementation of ZY68, Lohmann Animal Health GmbH & Co. KG, Cuxhaven, Germany; Declared activity: Endo-1,4-β-Xylanase (EC 3.2.1.8) 1000 FXU/g, ^a–h^ values with no common superscripts are significantly different within columns (*p* < 0.05), PSEM = pooled standard error of means.

**Table 6:** Blood serum clinical-chemical characteristics depending on dietary ergot, fat level and supplementation with a xylanase containing enzyme preparation (Day 14 of experiment, *n* = 8).

Group	Ergot (g/kg)	Soy Oil (g/kg)	Enzyme ^1^	Aspartate Aminotransferase (U/L)	Alanine Aminotransferase (U/L)	Glutamate Dehydrogenase (U/L)	γ-Glutamyltransferase (U/L)	Albumin (g/dL)	Total Bilirubin (µMol/L)	Antibody Titer to Newcastle Disease Virus ^2^
1	0	10	−	164.9 ^ab^	5.5 ^a^	6.5 ^a^	11.4 ^a^	1.6 ^a^	5.7 ^ab^	6.9 ^ab^
2	0	10	+	138.1 ^ab^	5.6 ^a^	6.3 ^a^	14.8 ^ab^	2.0 ^b^	5.7 ^ab^	6.3 ^a^
3	0	50	−	117.3 ^a^	6.6 ^a^	5.2 ^a^	12.9 ^ab^	1.9 ^b^	6.2 ^ab^	7.7 ^bc^
4	0	50	+	140.4 ^ab^	6.6 ^a^	6.1 ^a^	11.6 ^a^	1.7 ^ab^	4.7 ^a^	8.1 ^bc^
13	30	10	−	155.1 ^ab^	7.3 ^a^	10.8 ^b^	12.9 ^ab^	1.6 ^a^	6.7 ^b^	8.9 ^c^
14	30	10	+	183.6 ^b^	8.2 ^ab^	12.4 ^bc^	10.0 ^a^	1.9 ^b^	5.0 ^a^	6.8 ^ab^
15	30	50	−	177.4 ^b^	12.5 ^b^	8.9 ^ab^	18.5 ^b^	1.8 ^ab^	8.3 ^c^	8.8 ^c^
16	30	50	+	193.1 ^b^	9.2 ^ab^	15.4 ^c^	12.7 ^ab^	1.9^b^	6.2 ^ab^	7.8 ^bc^
*p-values*										
Ergot				<0.001	0.007	<0.001	0.454	0.905	0.017	0.003
Fat				0.712	0.074	0.886	0.145	0.358	0.149	0.001
Enzyme				0.273	0.608	0.012	0.145	0.041	0.002	0.003
Ergot × fat				0.040	0.366	0.452	0.033	0.635	0.051	0.112
Ergot × enzyme				0.196	0.611	0.032	0.019	0.528	0.122	0.007
Fat × enzyme				0.313	0.353	0.082	0.097	0.008	0.254	0.059
Ergot × fat × enzyme				0.092	0.374	0.258	0.706	0.263	0.527	0.875
PSEM				13.0	1.6	1.2	1.6	0.1	0.6	0.4

^1^ “−” without and “+” with supplementation of ZY68, Lohmann Animal Health GmbH & Co. KG, Cuxhaven, Germany; Declared activity: Endo-1,4-β-Xylanase (EC 3.2.1.8) 1000 FXU/g, ^2^ titer indicative figure, logarithm to the base 2, ^a–c^ values with no common superscripts are significantly different within columns (*p* < 0.05), PSEM = pooled standard error of means.

**Table 7:** Blood serum clinical-chemical characteristics depending on dietary ergot, fat level and supplementation with a xylanase containing enzyme preparation (Day 35 of experiment, *n* = 10).

Group	Ergot (g/kg)	Fat (g/kg)	Enzyme ^1^	Aspartate Aminotransferase (U/L)	Alanine Aminotransferase (U/L)	Glutamate Dehydrogenase (U/L)	γ-Glutamyltransferase (U/L)	Albumin (g/dL)	Total Bilirubin (µMol/L)	Antibody Titer to Newcastle Disease Virus ^2^
1	0	10	−	142.1 ^a^	8.4 ^bc^	4.0 ^abc^	23.1 ^ab^	2.1 ^c^	7.3 ^c^	8.1
2	0	10	+	167.3 ^ab^	9.9 ^c^	3.6 ^ab^	19.0 ^a^	1.8 ^b^	7.0 ^bc^	8.9
3	0	50	−	168.3 ^ab^	4.6 ^ab^	5.7 ^c^	29.9 ^b^	1.7 ^b^	5.0 ^a^	8.1
4	0	50	+	145.8 ^ab^	4.6 ^ab^	4.2 ^abc^	29.1 ^b^	1.9 ^bc^	6.0 ^abc^	8.5
9	10	10	−	162.9 ^ab^	4.3 ^ab^	3.3 ^a^	28.7 ^b^	2.1 ^c^	6.6 ^bc^	9.1
10	10	10	+	177.8 ^b^	2.8 ^a^	4.7 ^abc^	29.8 ^b^	1.7 ^b^	6.1 ^abc^	8.8
11	10	50	−	139.9 ^a^	4.1 ^ab^	5.4 ^bc^	20.6 ^a^	1.6 ^ab^	6.8 ^bc^	8.4
12	10	50	+	136.0 ^a^	3.8 ^ab^	3.6 ^ab^	30.4 ^b^	1.4 ^a^	5.5 ^ab^	8.3
*p-values*										
Ergot				0.771	<0.001	0.662	0.189	0.004	0.855	0.229
Fat				0.012	0.012	0.014	0.141	<0.001	0.023	0.055
Enzyme				0.559	0.937	0.083	0.330	0.001	0.541	0.337
Ergot × fat				0.004	0.003	0.373	<0.001	0.030	0.061	0.323
Ergot × enzyme				0.723	0.299	0.271	0.015	0.069	0.108	0.055
Fat × enzyme				0.006	0.947	0.002	0.058	0.004	0.746	0.796
Ergot × fat × enzyme				0.218	0.429	0.121	0.394	0.148	0.219	0.456
PSEM				8.2	1.1	0.5	2.2	0.1	0.6	0.3

^1^ “−” without and “+” with supplementation of ZY68, Lohmann Animal Health GmbH & Co. KG, Cuxhaven, Germany; Declared activity: Endo-1,4-β-Xylanase (EC 3.2.1.8) 1000 FXU/g, ^2^ titer indicative figure, logarithm to the base 2, ^a–c^ values with no common superscripts are significantly different within columns (*p* < 0.05), PSEM = pooled standard error of means.

**Table 8:** Organ weights (g/100 g live weight) depending on dietary ergot, fat level and supplementation with a xylanase containing enzyme preparation (Day 14, *n* = 8, and 35, *n* = 10, of experiment; note that only control groups and groups fed the highest ergot supplementation levels were slaughtered, meaning that for groups fed 30 g ergot/kg diet experiment was terminated at Day 14).

Group	Ergot (g/kg)	Fat (g/kg)	Enzyme ^1^	Heart		Spleen		*Bursa cloacalis*		Liver		Duodenum	Proventriculus + Gizzard	Pancreas
				d 14	d 35	d 14	d 35	d 14	d 35	d 14	d 35	d 35	d 35	d 35
1	0	10	−	0.782	0.497	0.085	0.102 ^ab^	0.355 ^a^	0.241 ^ab^	2.692 ^a^	2.343	2.946 ^c^	1.429^b^	0.205
2	0	10	+	0.751	0.515	0.068	0.106 ^abc^	0.291 ^ab^	0.274 ^b^	2.936 ^ab^	2.116	2.424 ^ab^	1.276^ab^	0.186
3	0	50	−	0.701	0.498	0.072	0.105 ^abc^	0.292 ^b^	0.276 ^b^	2.652 ^a^	2.157	2.543 ^b^	1.325^ab^	0.189
4	0	50	+	0.765	0.500	0.067	0.126 ^c^	0.268 ^b^	0.288 ^b^	2.843 ^a^	2.077	2.393 ^ab^	1.234^a^	0.186
13/9	30 (d 15)/10 (d 35)	10	−	0.828	0.478	0.088	0.093 ^a^	0.231 ^b^	0.255 ^ab^	3.362 ^bc^	2.143	2.328 ^ab^	1.271^ab^	0.191
14/10	30 (d 15)/10 (d 35)	10	+	0.756	0.483	0.092	0.098 ^a^	0.224 ^b^	0.212 ^a^	3.534 ^c^	2.071	2.244 ^a^	1.231^a^	0.201
15/11	30 (d 15)/10 (d 35)	50	−	0.853	0.476	0.081	0.121 ^bc^	0.245 ^b^	0.279 ^b^	3.075 ^abc^	2.026	2.365 ^ab^	1.382^ab^	0.199
16/12	30 (d 15)/10 (d 35)	50	+	0.773	0.483	0.081	0.112 ^abc^	0.269 ^b^	0.261 ^ab^	3.016 ^ab^	2.167	2.309 ^ab^	1.433^b^	0.207
***p-values***														
Ergot				0.075	0.069	0.063	0.502	0.007	0.235	<0.001	0.193	<0.001	0.805	0.350
Fat				0.828	0.743	0.210	0.007	0.761	0.048	0.041	0.262	0.211	0.446	0.927
Enzyme				0.312	0.511	0.542	0.383	0.400	0.781	0.227	0.277	0.003	0.283	0.905
Ergot × fat				0.353	0.821	0.877	0.423	0.087	0.699	0.139	0.351	0.045	0.038	0.367
Ergot × enzyme				0.121	0.877	0.324	0.195	0.211	0.084	0.475	0.087	0.046	0.244	0.242
Fat × enzyme				0.458	0.762	0.733	0.944	0.403	0.927	0.528	0.101	0.133	0.482	0.671
Ergot × fat x enzyme				0.379	0.717	0.536	0.198	0.919	0.453	0.693	0.761	0.195	0.897	0.600
PSEM				0.041	0.017	0.009	0.008	0.029	0.021	0.157	0.077	0.093	0.077	0.012

^1^ “−” without and “+” with supplementation of ZY68, Lohmann Animal Health GmbH & Co. KG, Cuxhaven, Germany; Declared activity: Endo-1,4-β-Xylanase (EC 3.2.1.8) 1000 FXU/g, ^a–c^ values with no common superscripts are significantly different within columns (*p* < 0.05), PSEM = pooled standard error of means.

**Table 9:** Apparent nutrient retention (% of intake) depending on dietary ergot and fat level (3rd week of experiment, *n* = 6).

Group	Ergot (g/kg)	Fat (g/kg)	Dry Matter	Organic Matter	Crude Fat	Nitrogen
1	0	10	67.6	70.1	82.6 ^a^	59.1 ^ab^
2	0	50	68.6	71.3	88.0 ^b^	59.2 ^ab^
9	10	10	66.1	68.9	82.5 ^a^	57.4 ^a^
10	10	50	68.6	71.0	88.9 ^b^	61.0 ^b^
13	30	10	69.6	72.4	84.1 ^ab^	61.5 ^b^
14	30	50	68.1	70.5	88.3 ^b^	62.0 ^b^
***p-values***						
Ergot			0.654	0.648	0.894	0.023
Fat			0.616	0.726	0.001	0.103
Ergot × fat			0.467	0.465	0.832	0.175
PSEM			1.6	1.6	1.6	1.0

^a–c^ values with no common superscripts are significantly different within columns (*p* < 0.05). PSEM = pooled standard error of means.

**Table 10:** Experimental design and further details.

Group	Ergot (g/kg Diet)	Total Ergot Alkaloids (mg/kg)	Soy oil (g/kg diet)	Enzyme ^1^	Growth Performance (*n* Pens with 8 Birds/Pen)		Clinical Chemistry, Organ Weights (*n*)		Ergot Alkaloid Residue Analyses (Blood, Bile, Liver, Breast Meat)	Intestinal Viscosity (*n* Samples Pooled from 10 Birds)	Balance Experiment (*n*)
					2 weeks	5 weeks	Day 14	Day v35		Day 35	Week 3
1	0	0.13	10	−	10	9	8	10	10	3	6
2	0	0.25	50	−	10	9	8	10	10	3	6
3	0	0.14	10	+	10	9	8	10	10	3	
4	0	0.08	50	+	10	9	8	10	10	3	
5	1	0.25	10	−	10	9				3	
6	1	0.25	50	−	10	9				3	
7	1	0.46	10	+	10	9				3	
8	1	0.28	50	+	10	9				3	
9	10	1.88	10	−	10	9		10	10	3	6
10	10	1.94	50	−	10	9		10	10	3	6
11	10	2.49	10	+	10	9		10	10	3	
12	10	2.03	50	+	10	9		10	10	3	
13	30	5.76	10	−	10		8				6
14	30	6.76	50	−	10		8				6
15	30	5.66	10	+	10		8				
16	30	5.85	50	+	10		8				

^1^ ZY68, Lohmann Animal Health GmbH & Co. KG, Cuxhaven, Germany; Declared activity: Endo-1,4-β-Xylanase (EC 3.2.1.8) 1000 FXU/g.
